# Missouri Citizen Perceptions: Giving Second Amendment Preservation Legislation a Second Look

**DOI:** 10.1017/jme.2023.39

**Published:** 2023

**Authors:** Kerri M. Raissian, Jennifer Dineen, Mitchell Doucette, Damion Grasso, Cassandra Devaney

**Affiliations:** 1: UNIVERSITY OF CONNECTICUT, HARTFORD, CT, USA; 2: JOHNS HOPKINS UNIVERSITY, BALTIMORE, MD, USA

**Keywords:** Second Amendment Preservation Act, Guns, Citizen Perception

## Abstract

In June 2021, Missouri passed the “Second Amendment Preservation Act” (SAPA). Though SAPA passed easily and had gubernatorial support, many Missouri law enforcement agencies, including the Missouri Sheriff’s Association, oppose it. Missing from this policy conversation, and deserving of analysis, is the voice of Missouri citizens. Using qualitative interview data and survey data, we explored what if anything Missouri gun owners knew about SAPA and what they perceived its effects would be on gun-related murders, suicides, gun thefts, and mass shootings. Most Missouri gun owners had not heard about SAPA and were ambivalent about its potential effect on gun safety outcomes. Our findings also indicate that respondents’ attitudes toward SAPA and the impact of such policy on safety is driven by gun ownership (i.e., primary versus living in a household with firearms), partisan identification, and attitudes toward government firearm regulation.

## Introduction

In the United States, gun violence remains a persistent public health issue that has regrettably progressed to the leading cause of death for children and adolescents.^1^ In response, and towards preventing gun violence, states such as California, Connecticut, Massachusetts, and New York have established restrictive policies that — broadly speaking — regulate who has access to firearms and where firearms can be used (e.g., Extreme Risk Protection Orders [ERPOs], minimum age requirements, and Child Access Protection [CAP] laws). In stark contrast, states such as Arizona, Idaho, Kansas, Missouri, and Wyoming have responded with a permissive policy that minimizes restrictions on gun ownership and expands permissible use (e.g., Right-to-Carry, Stand-Your-Ground, and removing or forgoing ownership restrictions).

Perhaps one of the most permissive types of laws is Missouri law HB85, also known as the “Second Amendment Preservation Act” (SAPA), which declares federal regulations that restrict gun ownership among state citizens invalid. It prohibits public officials from “enforce[ing] any laws, rules, orders, or actions that violate the Second Amendment rights of Missourians.”^2^ Though SAPA generated much public debate and controversy among public officials,^3^ citizen perception of SAPA remains missing from the policy debate and conversation. Given that SAPA — and laws like it — are intended to promote public safety, via allowing citizens greater access to their personal firearms to prevent and defend a personal attack, we ask how Missouri citizens perceive the effect of SAPA. To date, it is not known how Missouri citizens — those most affected by SAPA’s passage — may perceive the impact of SAPA on their safety and negative, gun-related outcomes, such as gun-related murders, suicides, mass shootings, and gun theft.

The literature lacks research that investigates the implementation or impact of Second Amendment sanctuary on citizens’ perceptions of safety. As such, we sought to understand the attitudes of gun-owning Missouri citizens towards the SAPA law passed in 2021. We aimed to identify the extent to which gun-owning Missouri citizens are aware of SAPA, the extent to which these Missouri citizens think SAPA will make their state safer or more dangerous, and the extent to which gun-owning Missouri citizens think SAPA will reduce or increase murders, suicides, gun thefts, and mass shootings.

The literature lacks research that investigates the implementation or impact of Second Amendment sanctuary on citizens’ perceptions of safety. As such, we sought to understand the attitudes of gun-owning Missouri citizens towards the SAPA law passed in 2021. We aimed to identify the extent to which gun-owning Missouri citizens are aware of SAPA, the extent to which these Missouri citizens think SAPA will make their state safer or more dangerous, and the extent to which gun-owning Missouri citizens think SAPA will reduce or increase murders, suicides, gun thefts, and mass shootings. Although our paper focuses on Missouri, the following section will note that within the United States, both state and local levels of government have adopted SAPA and SAPA-related legislation. Therefore, in addition to exploring gun-owning Missourian’s perceptions of SAPA, this study may inform the need to incorporate citizen perception into gun legislation more broadly.

The paper proceeds as follows. First, we provide a brief overview of federal firearm policy and SAPA or SAPA-related laws. We then describe our methods and present results. Lastly, we discuss the implications of this work, note limitations, and offer concluding statements.

## Brief History of Federal Firearm Policy and SAPA Laws

In the absence of robust federal firearm policy,^4^ states’ policy responses^5^ have largely been the primary way firearm regulations have occurred in the U.S. States have self-selected into either “restrictive” gun law states, where the scope and number of laws seek to control how firearms are obtained and who is legally allowed to obtain them (i.e. restricting gun access and often referred to as “gun control”), and “permissive” gun law states, where there is an absence of regulatory laws or explicit expansion of gun access.^6^ Research has noted that states with restrictive gun laws typically have lower rates of firearm homicides, firearm suicides, and other negative health outcomes.^7^

Gun rights organizations like the National Rifle Association (NRA) have opposed restrictive gun policy at the state and federal level,^8^ even in the wake of devastating mass shootings at Columbine High School and Sandy Hook Elementary. Concurrently, many states have preempted local firearm laws. A recent analysis of state-level firearm laws and preemption found that most states have local preemption measures on almost all gun-control policy topics, including assault weapon bans, while choosing not to enact state-level gun control measures. In addition, from 2009-2018 the number of states with punitive preemption measures doubled, which would allow state attorney generals to sue local governments or local officials for passing local gun ordinances.^9^

This local legal environment along with the lack of sustained federal movement on firearm policy made it possible for several localities and states to declare their jurisdictions as Second Amendment sanctuary cities. These “sanctuary cities,” starting with Effingham County, Illinois in 2018,^10^ effectively declared that they would refuse to enforce and dedicate resources to the implementation of restrictive gun measures — specifically those mandated by the federal government.

Today ten states have state-level sanctuary legislation.^11^ In some states the legislation appears symbolic and objects to any infringement of Second Amendment rights. In others (like Missouri), the sanctuary legislation explicitly states that laws deemed — presumably by the local officials — to present unconstitutional restrictions on the Second Amendment will not be enforced and fines are imposed on individuals that attempt to enforce “unconstitutional” laws.^12^

Proponents of Second Amendment sanctuary laws praise the legislation as a legal shield from government overreach. Opponents, however, argue that Second Amendment sanctuary laws can have devastating consequences for individuals trying to use said laws as a defense and create legal and ethical dilemmas for law enforcement officials who have a duty to enforce these laws.^13^ The Second Amendment sanctuary laws make the validity and enforceability of federal firearm regulation unclear to local and state law enforcement, which may frustrate inter-jurisdictional efforts to address crime.^14^

In June of 2021, Missouri followed several other states^15^ to pass its own Second Amendment sanctuary law, referred to as the Secondary Amendment Preservation Act (SAPA),^16^ which prohibits state agencies from helping the federal government to enforce any law, rule, or regulation that the Missouri state government considers an infringement on the right to bear arms.^17^ The law aims to invalidate federally mandated gun laws in the state by declaring “…as invalid all federal laws that infringe on the right to bear arms under the Second Amendment to the U.S. Constitution.”^18^ The law purports to protect Second Amendment rights, stating that “federal supremacy does not apply to federal laws that restrict or prohibit the manufacture, ownership, and use of firearms, firearm accessories, or ammunition within the state because such laws exceed the scope of the federal government’s authority.”^19^ Furthermore, the law prohibits the use of Missouri state resources from being used to further federal gun legislation and criminalizes the enforcement of any firearm laws declared invalid under the Act. The bill dictates under what circumstances a Missouri law enforcement officer can help a federal officer investigate or prosecute a gun crime; violations can carry a $50,000 civil penalty. In response, the Department of Justice sued Missouri to block the law.^20^ The suit is currently pending a verdict.

Missing from the conversation is understanding how citizens view Second Amendment sanctuary laws and, specifically, how citizens perceive the potential these laws have to impact public safety. Most states, including Missouri, lack a straightforward mechanism for citizens to have direct input into policy.^21^ Citizen perception of firearm policy and the impact of such policy on personal and public safety is important for driving policy development and implementation. Research has demonstrated associations between gun ownership and perceptions of safety risk^22^ and negative outcomes, such as violent crime, murder, and suicide.^23^ Citizen perception of SAPA on personal and public safety could affect gun-related behaviors such as ownership and carry choices and, in turn, affect related injury and death rates.

## Methods

To inform our investigation of citizen stakeholder attitudes regarding the impact of Second Amendment preservation policy on safety and outcomes (e.g., murder, suicide, and mass shootings) we conducted qualitative interviews with a sample of Missouri gun owners, as well as a national survey of U.S. adults, including an oversample of Missouri residents.

### National Survey

The current nationally representative sample of Americans ages 18 and older was established from the SSRS probability panel and included an Ipsos Knowledge Panel oversample of Missouri households with a gun. The SSRS Probability Panel is a mixed-mode representative panel, which is generalizable to the U.S. population. SSRS Probability Panel members are recruited randomly from a dual-frame random digit dial (RDD) sample via the SSRS Omnibus survey. Those recruited without internet access were contacted via telephone to complete the survey. The IPSOS Knowledge Panel is randomly recruited using probability sampling. Households are provided with internet access when needed. Data collection occurred between April 21 and May 15, 2022, and this yielded a sample of 181 Missouri adults within the larger sample of 2,007 Americans. After removing respondents who did not own guns (*n* = 10) and those with missing data (*n* = 2), our final analytic sample was 169 Missourians who lived in a home with a gun.

The survey was developed by the Johns Hopkins Center for Gun Violence Solutions and the University of Connecticut. Survey data collection vendor, SSRS, initially reviewed the questionnaire with the goal of identifying potential problems related to respondent burden, item and unit non-response, respondent comprehension, and practical challenges related to survey mode. SSRS feedback informed several iterations of revisions. Survey questions address how citizen stakeholders perceive firearm policy (specifically in the areas of safe storage and open carry) as being beneficial or harmful, citizen relationships with firearms (i.e., ownership, types of use, reasons for owning/not owning, if and where they open carry), and several demographic variables. We know if the respondent is the primary gun-owner (i.e., they are the legal owner of the gun), or if the respondent simply lives with the primary gun-owner (i.e., the secondary owner of the gun). Respondents from Missouri additionally received questions investigating their awareness of and attitudes towards the SAPA, as well as its perceived impact on firearm murders, suicides, gun thefts, and mass shootings.

Web panelists were emailed an invitation to complete the survey online. Panelists who did not respond to the email invitation received up to two reminder emails, and panelists who had opted into receiving text messages from the SSRS Opinion Panel received two text message reminders. SSRS panelists without web access were contacted via telephone. Interviews were completed in English or Spanish using a CATI system. To maximize survey response, up to 10 attempts were made to selected numbers. The survey completion rate was 44.9% (Completions/Total invited to participate). The Methods Appendix, provides the distribution of sample characteristics as they occur in the population, final sample, and final sample weighted to adjust for non-response.

### Qualitative Interviews

As part of survey development, and to ensure survey items were adequately operationalized and relevant in assessing perceived harms and benefits associated with a given firearm policy, the research team conducted cognitive pre-tests with 30 Missouri gun owners. Missouri cognitive pre-test respondents were recruited via a random telephone sample for a 60-minute interview. Interviews were conducted by Stavisky and Associates. The qualitative interviews conducted via phone and Zoom, between January 6 and February 18, 2022, were recorded and transcribed. The final section of the cognitive pre-test portion of the interview posed open-ended interview questions inquiring about the meaning of gun ownership, understanding of restrictive gun policy, especially Child Access Prevention laws, and degree of support for the Second Amendment (generally) and SAPA. Complete information regarding the project methodology, including wording for relevant survey questions, is presented in the Methods Appendix.

## Analysis

Our national sample contained 181 Missouri respondents. However, due to sample composition, we removed the 10 respondents who did not own guns or live in a household with someone who owns a gun. Two additional respondents were missing income covariate data, and so they were also removed from our analytic sample of 169 Missourian adults.

### Outcome Measures

We asked Missouri respondents six distinct SAPA related questions to understand Missouri residents’ knowledge of SAPA and perceptions of the policy’s effect on various public safety measures. Our SAPA specific questions were preceded by the following description of the policy: “Last year, Governor Parson signed the ‘Second Amendment Preservation Act’ into law. The Act prohibits state agencies from helping federal officials enforce any laws or rules that the State of Missouri believes violate Second Amendment rights.” We then asked Missouri respondents the following questions:

1. How much have you heard or read about the Second Amendment Preservation Act in Missouri? (1) Nothing at all, (2) Only a little, (3) Some, or (4) A great deal.
2. In general, do you think the Second Amendment Preservation Act makes Missouri: (1) Less Safe, (2) Has No Impact on Safety, or (3) More Safe?

For questions 3-6 below, respondents were able to answer using a 5-point scale, with the following designations: (1) Significantly decrease, (2) Somewhat decrease, (3) no effect, (4) Somewhat increase, and (5) Significantly increase.

1. To what extent, if any, do you think the Missouri Second Amendment Preservation Act can impact the number of people murdered by guns?
2. To what extent, if any, do you think the Missouri Second Amendment Preservation Act can impact the number of people who use a gun in suicide?
3. To what extent, if any, do you think the Missouri Second Amendment Preservation Act can impact the number of guns stolen each year?
4. To what extent, if any, do you think the Missouri Second Amendment Preservation Act can impact the frequency of mass shootings?

### Analytic Approach

We used two distinct but related analytic strategies to address research questions. First, using descriptive analyses, we explored how awareness, support, and the perceived impact of SAPA varied among Missouri residents. These analyses also compared the attitudes of Missouri gun households to attitudes of gun households in other states with significant Second Amendment preservation efforts, including Arizona, Arkansas, Idaho, Kansas, North Dakota, South Dakota, Tennessee, Texas, and Wyoming (henceforth other SAPA states), and U.S. states without significant SAPA efforts (henceforth, non-SAPA states).

We first ascertain if Missouri citizens living in a household with a gun are aware of SAPA and if they believe SAPA will change gun related murders, suicides, gun thefts, and mass shootings. We examine the outcome distributions for each of our six aforementioned survey items. In addition to examining the outcome distribution for the entire sample, we also examine the outcome responses by select, demographic characteristics such as education, gender, and race; political variables such as party identification and attitudes toward government regulation, and gun ownership versus non-owners living in gun households. Detailed question wording for each measure is presented in the Methods Appendix.

Next, we examine predictors of Missourians’ knowledge and beliefs regarding SAPA, using ordinary least squares (OLS) regression.^24^ Specifically, we estimate the following equation:

(1)

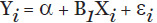




Where Y corresponds to each of the SAPA specific outcome measures for each respondent *i*. We will present results from Equation 1 with three different model specifications.

Our first model specification regresses a set of baseline demographic characteristics on our six SAPA outcome measures. These baseline measures include dummy variables that control for the respondent’s race and ethnicity (white, non-Hispanic; Black, non-Hispanic; other, non-Hispanic; and Hispanic), the respondent’s highest educational attainment level (high school or less, some college, or college and beyond), the respondent’s political party self-identification (Republican, Democrat, or Independent), and the respondent’s religious characterization (not religious, slightly to moderately religious, and very religious). We also include household income categories (household income is under $25,000; $25,000-$49,000; $50,000-$74,000; $75,000-$99,000; $100-$124,000; $125,000-$149,000; $150,000-$174,000; $175,000-$199,000; $200,000 or higher), and the respondent’s age group (18-29; 30-49; 50-64; and 65 plus).

While we do not know where in Missouri respondents live, SSRS includes a population density variable that ranges from 1 (least dense quintile) to 5 (most dense quintile). We include this density variable to control for the respondent’s urbanicity. We also asked respondents about their prior victimization:

1. At any time in your life: Has someone ever physically assaulted or hit, punched, shoved, choked, kicked, or hurt you?
2. At any time in your life: Has someone ever threatened to harm you or someone you care about with a firearm?

From these questions, we created two distinct indicator variables to address the respondent’s own safety and prior victimization within their lifetime. If respondents indicated they had been physically assaulted, they were coded as a 1 (0 otherwise). If respondents indicated they or a loved one had been threatened with a firearm, they were coded as a 1 (0 otherwise).

We also measured respondents’ support of government intervention to regulate how guns are handled, for which 0 represented complete opposition and 10 complete support. Some categories had very small samples and so we collapsed the variable to conform to a 3-point scale; respondents answering 0 to 3 opposed additional government regulation of how firearms are handled, respondents answering 4 to 6 were neutral about government regulation of how firearms are handled, and respondents answering 7 to 10 supported government regulation of haw firearms are handled.

Model specification 2 maintained all covariates in the baseline specification (as described above) but included a measure to account the respondents’ gun ownership status. All respondents in our survey live in gun owning households, but we distinguished between respondents that personally own a gun (i.e., the gun in the household belongs to them) and secondary gun-owners (i.e., those that live in a household with the primary gun-owner).

Finally, model specification 3 builds on model specifications 1 and 2 by explicitly controlling for whether the respondent has heard of SAPA. While model specifications 1 and 2 treated this variable (if the respondent has heard of SAPA) as an outcome variable, model specification 3 will use this measure as an explicit independent control to test whether information about the policy impacts respondent’s beliefs about how the policy will impact gun-related outcomes.^25^

## Results

### Descriptive Statistics

Table 1 presents descriptive statistics. The first rows of Table 1 display the means and standard deviations for the outcome variables in our study. These questions are unique to Missouri’s SAPA law, and thus were only asked of Missouri respondents. On average, the Missouri sample reported that they have not heard of SAPA, and respondents tended to report that SAPA would lead to an increase in overall safety. However, when asked about specific types of gun related safety outcomes, Missouri respondents tended to report that SAPA would increase murders, suicides, and gun thefts. They also tended to report that SAPA would not affect the frequency of mass shootings.

**Table 1:**
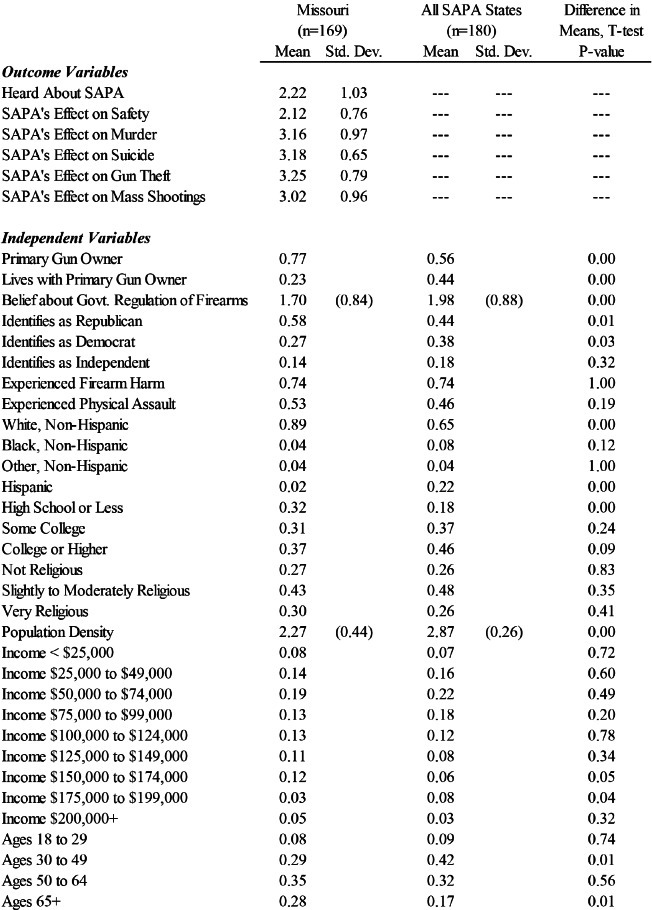
Descriptive Statistics

Table 1 describes two samples: our analytic sample of Missouri gun-owners and a comparison sample of gun-owners in other SAPA states (Alaska, Arizona, Idaho, Kansas, North Dakota, South Dakota, Tennessee, Texas, and Wyoming).^26^ The first columns present means and standard deviations (for continuous variables) for both outcome and independent variables for the Missouri gun-owner sample. Among Missouri gun owners, most gun owners (77%) identified as the primary gun owner, were neutral on whether or not government should regulate how firearms are handled, identified as Republican (58%), and were White (89%). Most of the Missouri respondents identified as being religious, and about 68% of the sample reported having an education beyond high school. Seventy-four percent of the Missouri sample has been or has had a loved one threatened by a firearm, and about 53% reported having experienced physical assault.

In the lower portion of Table 1, Columns 3 and 4 present the same descriptive statistics^27^ for other SAPA states, and Column 5 presents the p-value from a T-test of mean difference. Overall, Missouri is somewhat distinct from the other-SAPA states. Missouri gun-owners are more likely to identify as the primary gun owner (77% versus 56%), are less likely to support government regulation of firearms (1.70 versus 1.98), are more likely to identify as Republican (58% versus 44%), and more likely to be White, Non-Hispanic (89% versus 65%). Although respondents in the Missouri sample are more likely to live in a rural area, they are just as likely to have experienced a threat with a firearm and physical assault. These differences should be kept in mind when interpreting the results and when considering generalizability of the study to other states or settings.

### Outcome Distributions

**Figure 1:**
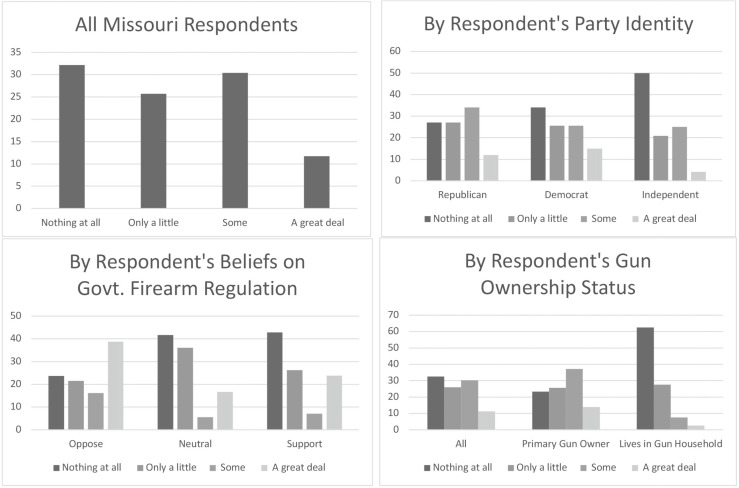
Distribution of Having Heard of SAPA, by Select Independent Variables

To better understand the average responses of the six outcome variables, we present each outcome’s distribution across select demographic variables. We examined whether or not each outcome measure varied by the respondents’ race and ethnicity, party identification, belief about the government’s role in firearm regulation, and the respondent’s gun ownership status. These distributions are displayed in Figures 1 through 6 — each containing a set of 4 bar graphs.

**Figure 2:**
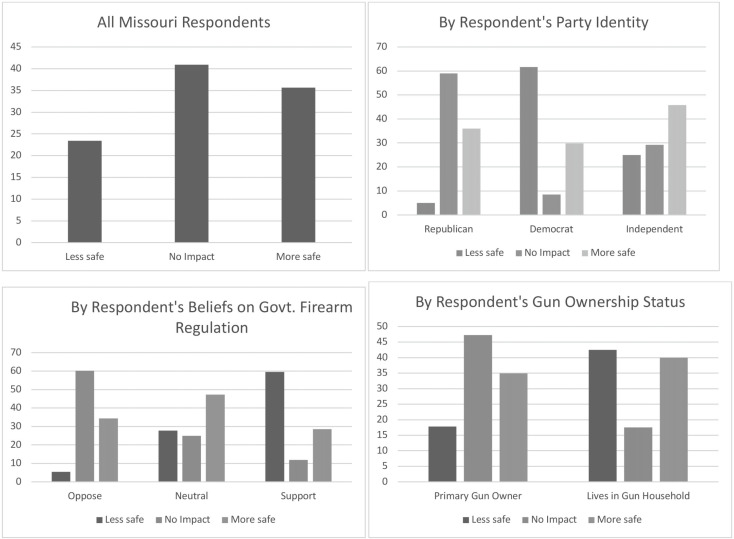
Distribution of Respondent’s Belief of SAPA’s Impact on Safety, by Select Independent Variables

**Figure 3:**
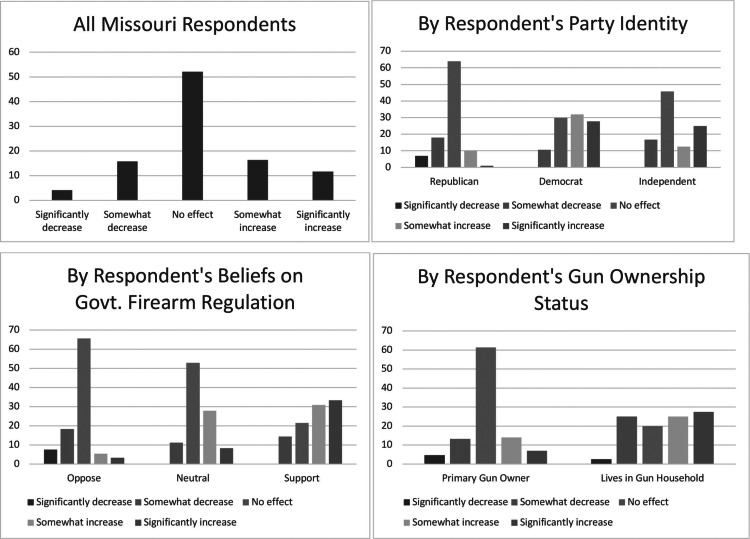
Distribution of Respondent’s Belief of SAPA’s Impact on Murder, by Select Independent Variables

**Figure 4:**
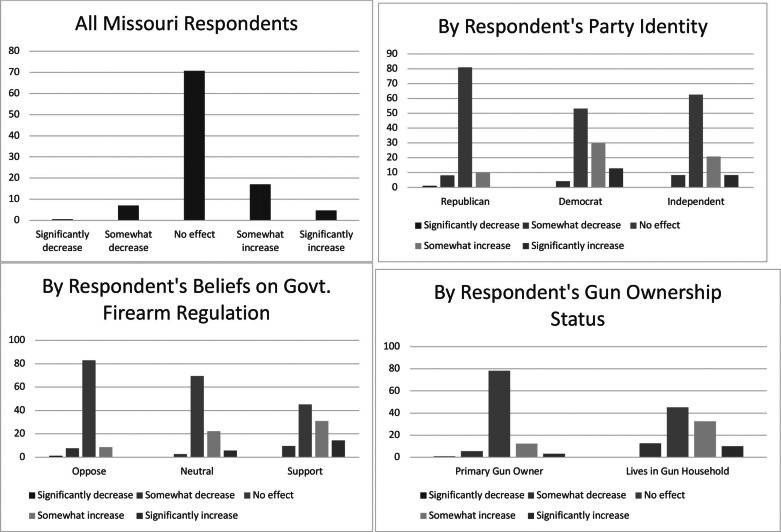
Distribution of Respondent’s Belief of SAPA’s Impact on Suicide, by Select Independent Variables

**Figure 5:**
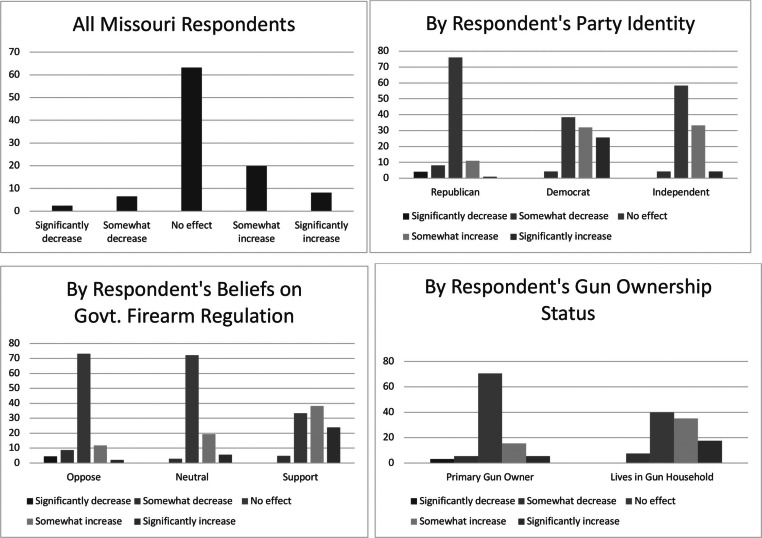
Distribution of Respondent’s Belief of SAPA’s Impact on Gun Theft, by Select Independent Variables

**Figure 6:**
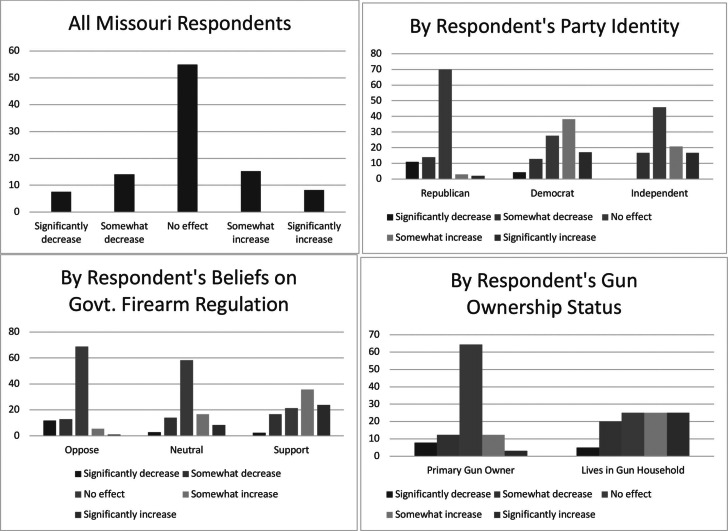
Distribution of Respondent’s Belief of SAPA’s Impact on Mass Shooting, by Select Independent Variables

Figure 1 shows the distribution of respondents having heard of SAPA. Although the mean of this variable is 2.22, indicating on average, respondents have heard “only a little” to “some” about SAPA, the distribution shows that the modal response is “nothing at all.” We also examine this distribution by party affiliation, the respondent’s belief that government should regulate firearms, and the respondent’s ownership status. Of the sample, Republicans, individuals opposing gun regulation, and primary gun owners are all most likely to have heard about SAPA.

Figure 2 shows the distribution of respondents’ belief that SAPA will impact safety. Consistent with the 2.22 mean, most gun-owning Missourians tend to report that SAPA will not impact safety. However, this perception is not shared by all Missourians in our sample. Overwhelmingly, Republicans, individuals opposing firearm regulation, and primary gun owners more often report that SAPA will have no impact on safety, though when these groups do anticipate SAPA could influence safety, they are more likely to believe SAPA will increase rather than decrease safety. In contrast, Democrats, those that support government regulation, and those that simply live in a household with a firearm are much more likely to believe SAPA will lead to decreased safety.

We next explored specific types of safety outcomes that SAPA might impact; i.e., gun-related murders, suicides, gun thefts, and mass shootings. Figures 3 through 6 show the distribution of each outcome by the same select characteristics. Similar patterns emerge across all safety measures. Republicans, individuals opposing gun regulation, and primary gun owners tend not to think that SAPA will affect gun related murders, suicides, gun thefts, or mass shootings, and in all outcomes except mass shootings, they lean slightly towards expecting fewer negative outcomes. Similar to the aforementioned groups, non-Republicans, individuals supporting increased gun regulation, and non-primary gun owners tend to report that SAPA will have no effect on these measures of safety outcomes, *but* they appear much more likely to anticipate an increase in murders, suicides, and thefts following SAPA’s adoption.

### Regression Models

Table 2 presents results from OLS models. The first columns of Table 2 present the results for characteristics that are associated with the respondent having heard of SAPA. Model specification 1 results suggest that older males with more than a high school education tend to have heard of SAPA, while controlling for full set of covariates described in the baseline model above. In model specification 2, we also include primary gun owner as an independent variable, and this is significantly associated with having heard or read about SAPA. Meanwhile, the previous respondent characteristics attenuate slightly (though remain significant). Notably, respondents’ beliefs regarding government intervention is not significant in any of the model specifications.

**Table 2:**
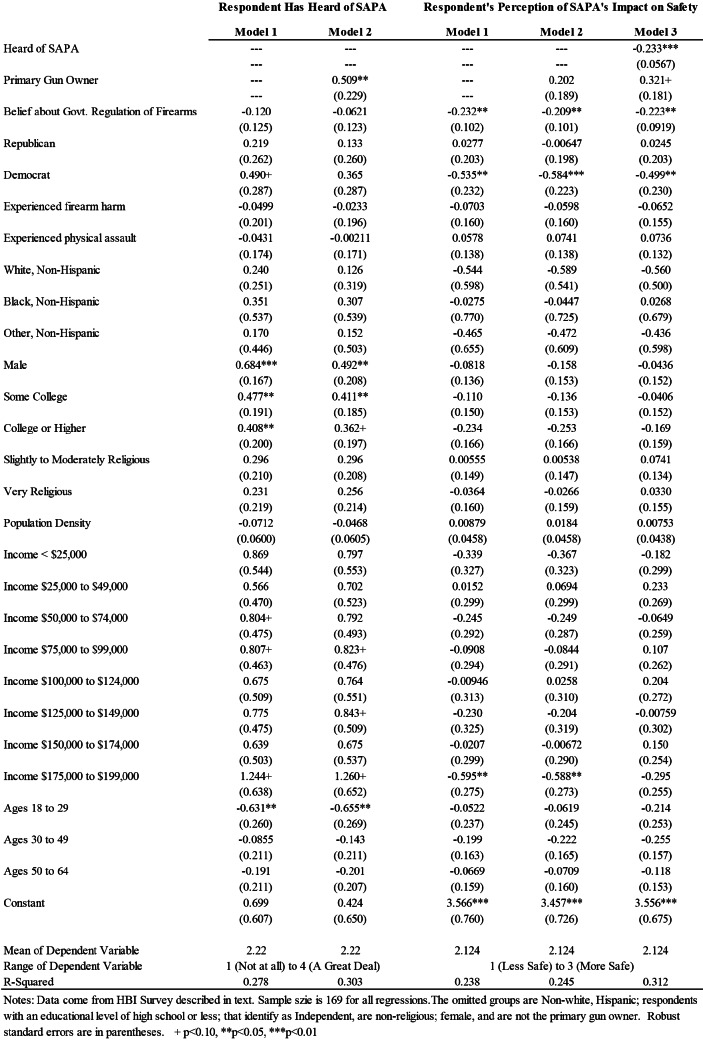
OLS Regression Results Regarding SAPA Policy

Columns 3-5 of Table 2 present results of Model Specifications 1-3 in predicting respondents’ perception that SAPA will impact safety. In the first model specification, if a respondent supports government intervention or identifies as a Democrat, they tend to report that SAPA will reduce safety. In model speciation 2, we include primary gun owner as an independent variable, which does not change the significance of the results. Finally, model specification 3 includes whether a respondent has heard of SAPA, which is significantly associated with increased tendency to report that SAPA will reduce safety.

Next, we probe how characteristics are associated with respondents’ belief that SAPA will impact measures of safety, namely gun related murders (columns 2-4), suicides (columns 5-7), thefts (columns 8-10), and mass shootings (columns 11-13). Across all model specifications, one variable emerges as a key predictor of respondents’ perception that SAPA will increase gun related murders, suicides, gun thefts, and mass shootings. If the respondent supports government regulation of firearms, they believe that SAPA will increase each of these outcomes (gun related murders, suicides, gun thefts, and mass shootings), thereby eroding safety. Other variables also emerge as significant predictors of specific outcomes. For example, individuals who identify as Republicans tend to report that SAPA will decrease murders and mass shootings, and primary gun owners tend to report that SAPA will decrease gun thefts. Having heard of SAPA is never significantly associated with respondents’ perceptions regarding SAPA related outcomes.

### Qualitative Interviews

The quantitative results suggest that Missouri gun owners opposing government regulation of firearms are more likely to believe that SAPA will enhance safety via reducing gun murders, suicides, gun thefts, and mass shootings. However, these results are best understood with some context. Therefore, we next provide excerpts from the qualitative part of our study that serve to illuminate Missouri gun owners’ thoughts about government gun regulation of firearms.

Consistent with our quantitative results, some gun owners believe that additional gun regulation is not needed, with one respondent commenting, “We don’t need any more regulations cuz it will be regulated to the point you can’t use it [firearm].” Another respondent remarked, “I have that [gun owning] right as an American. I’m proud that I have that 2nd Amendment right [to a gun]. Other countries don’t have that.” And we heard from another respondent that “There is a never-ending questionnaire of how heavily the government should be involved in regulating guns. The government takes over, but they have no right to, it’s [owning a firearm is] guaranteed in the 2nd amendment.”

As to the issue of who — or which level of government — should decide matters of gun regulation, one respondent told us, “In my mind, the federal government needs to stay out of state stuff.” This sentiment implies that government at the state-level is in a better position to determine the proper regulations to keep citizens at the state-level safe.

However, we also heard from several respondents about what they felt was an appropriate level of government regulation of firearms. In particular, “Initially, I believe that any American can possess a gun, until that right is taken away from them by law. Whether they’re a criminal or have a mental disability that should prevent them from being able to possess one.” And another respondent remarked, “I don’t want people to take that [gun owning] right away. I have that right, but it’s more of a privilege… it’s like driving a car. If you don’t follow the law, you lose it.” And yet another person told us, “…it’s in the Constitution. I’m very opinionated about that part of the Constitution. That’s every American law-abiding citizen’s right… There are unlawful Americans that have committed crimes and should lose that right. Gun ownership is a privilege, such as driving. It has to be a law-abiding citizen. If it applies to everyone there are no laws to be enforced.”

Finally, at least one respondent took a more nuanced view to the Constitution. They told us, “The biggest issues – how much background check is enough background check and the 2nd amendment. Do those words say exactly what a lot of people take from them? People that think unlimited firearm ownership is protected by the 2nd amendment fail to recall the part that talks about militias. They’re not taking into consideration when that document was written. I had a co-worker tell me, we were having a discussion and he says, “I guarantee you the word ‘machine gun’ is in the constitution.” He believed it was a constitutional right to own a machine gun.”

Thus, while the future and legality of SAPA remains unclear, what is clear is the need for citizens to be thoughtfully engaged on laws which directly affect their Second Amendment rights and their everyday safety.

## Limitations

Even when employing rigorous methodology to obtain a nationally representative, probability-based sample, a cross-sectional survey has limitations. To produce clear and broadly applicable measures, standardized surveys force a researcher to simplify complex constructs. Future research may include investigating how perceptions of safety will inform firearm behavior.

Cross-sectional studies can provide a detailed picture of an issue and population, but such studies are limited by being carried out at a single time point. Due to the lack of temporal variation, this single survey does not provide evidence of causation. Related are the challenges due to a limited time researchers can keep respondents engaged. Thus, in addition to not being able to ask all the desired questions, for some data, there is a lag between when variables were measured (i.e., demographic variable collected during panel recruiting and refreshing) and the March and April 2022 survey data collection.

Using Missouri as a case study, this study provides a critical glimpse into how Americans in gun owning households within a permissive firearm policy environment perceive the potential impact of SAPA, an extremely permissive gun policy, on public safety. We find a disconnect between policymaking and citizen engagement.

The survey used in this paper is part of a larger, national study. Therefore, our resources for collecting a Missouri oversample were limited. Our analytic sample of 169 gun-owners in Missouri allowed us to examine their perceptions of SAPA on safety, but the precision of our estimates and ability to analyze subgroups are limited by the sample size. Our sample also lacked a sufficient number of non-gun owning Missouri households to facilitate comparison. While this study is able to examine non-gun owners in other SAPA states, future state-specific work should include non-gun owing households to improve estimates of their perceptions on safety and the potential impact on behavior.

## Discussion

Using Missouri as a case study, this study provides a critical glimpse into how Americans in gun owning households within a permissive firearm policy environment perceive the potential impact of SAPA, an extremely permissive gun policy, on public safety. We find a disconnect between policymaking and citizen engagement. Despite the robust discussion of SAPA among policymakers, advocates, and scholars, the most common response from survey respondents was that they had not heard of the law. Primary gun owners had heard more about SAPA than secondary gun-owners (and as they were not in our sample, we do not know the extent to which citizens in Missouri’s non-gun owning households have or have not heard of SAPA). Yet all citizens, primary gun owners, secondary gun owners, and non-gun owners,^28^ should be involved in the policy discussion process.

Moreover, as it relates to overall safety and each gun safety outcome — the modal (or most frequent) response among Missouri gun owners was that SAPA would not affect overall safety or particular gun-safety outcomes. When we analyze the mean response (rather than the mode), we find a divergence between Missouri gun owners’ perceptions of SAPA’s general impact on safety and how the law will impact specific outcomes. While Missourians lean slightly toward believing SAPA will increase overall safety, they also lean towards believing SAPA will increase gun murders, suicides, and theft. As it relates to mass shootings, Missouri gun owners tend not to believe SAPA will have an impact. This is interesting, as mass shootings such as Sandy Hook, Las Vegas, Parkland, and more recently, Uvalde, each served as a catalyst for policy change.

Results also suggest that SAPA awareness and perceptions of safety do not vary by standard demographics such as race, income, education, and gender; however, they do vary by respondents’ partisan identification and feelings about government firearm regulation. Respondents who identify as Republican, primary gun owners, and individuals opposing government regulation of firearms tend to report higher levels of SAPA awareness (having heard about SAPA) and are most likely to believe SAPA will have no impact on overall safety or most specific gun-related outcomes.

Regression models are consistent with the bivariate analysis. We find partisanship and belief toward government regulation as significant predictors of the perceived impact of SAPA on safety, controlling for several demographic characteristics. We found that Democrats along with those who support government regulation believe SAPA will decrease safety. When examining characteristics associated with beliefs that SAPA will impact measures of safety, namely gun related murders, suicides, thefts, and mass shootings, we found support of government regulation of firearms to be the one consistent predictor associated with the respondent’s belief(s) that SAPA will increase those outcomes, thereby decreasing safety.

Our findings indicate that respondents’ attitudes toward permissive gun policy and the impact of such policy on safety is driven by gun ownership status (i.e., primary versus secondary gun owners), partisan identification, and attitudes toward government firearm regulation. These findings manifest in a myriad of ways — both in politics and in everyday life.

We underscore that the usual justification for permissive gun laws is that they will promote public safety via allowing for easier defensive gun use. However, it seems that Missouri gun owners themselves are not convinced that SAPA laws will facilitate a reduction in our measures of safety. Future work should consider why legislators enact these laws — is it to reduce negative gun related outcomes, a belief in the Second Amendment irrespective of public safety, political posturing, or something else?

Politically, policy positions that are driven by political leanings rather than knowledge or awareness are difficult to change or alter.^29^ Prior work finds partisanship to be a predictor of some gun control policy attitudes^30^ and points to relatively recent partisan polarization to explain policy gridlock. This relationship between partisan identification and policy attitudes is thought to reduce the opportunity for compromise across issues and may strengthen affective ties to partisan identities, which in turn makes bipartisan policy less likely.^31^ Partisanship driving attitudes toward gun policy are problematic as the gap in views of Democrats and Republicans on gun policy is wider than it is regarding most other policy issues.^32^ This gap points to significant hurdles passing legislation that might effectively reduce gun violence and injury. Further, bipartisanship increases overall legislative effectiveness and moving legislation through committee to the floor.^33^ It is difficult for lawmakers seeking re-election to engage in bipartisan policy efforts if their constituents are highly polarized and not informed of pending or newly elected legislation.

The impact of polarization of citizen attitudes toward firearm policy, especially related to licensure, storage, child access, and acquisition also impacts the extent to which policy implementation can be successful. These policies rely on citizens’ willingness to purchase, store, and license firearms in specific ways that may not be the most convenient or may require them to incur costs (i.e., locking your gun in a safe, applying and paying for a license). Policy perceived as grounded in politics, rather than best practice for safe use, may not be well received and implemented by the citizens we depend on to do so.

Because messaging surrounding gun laws is primarily partisan and information levels about policy is low, there is potential for dangerous confusion. The recent push by states such as Kansas, Wyoming, and Texas to enact similar SAPA laws and nullify federal policy, along with the recent Supreme Court decision in *New York State Rifle & Pistol Association v. Bruen*, have made the standing of various gun laws unclear — potentially and especially for the average citizen.^34^ This lack of clarity can lead citizens to unknowingly engage in illegal behavior(s). For example, after the 2013 passage of SAPA legislation in Kansas, some residents believed they could legally sell Kansas-made silencers without federal oversight so long as the silencers stayed in-state.^35^ The nuance of SAPA law, that it is a commitment of the state not to enforce federal law rather than nullify federal law, may not be obvious to many citizens.

At the time of this paper’s writing, Missouri’s SAPA law is being challenged both by the United States Department of Justice as well as by St. Louis and Jackson County. In both instances, the plaintiffs argue that SAPA violates the Supremacy Clause of the United States Constitution (that is that federal law supersedes any conflicting state law). These lawsuits are occurring in a broader judicial context, with the Supreme Court declaring in June 2022 that New York’s “may issue” firearm permitting structure unconstitutional. The Court’s majority opinion written by Justice Thomas indicated laws infringing on Second Amendment rights must have widely accepted historical analogues. If the above lawsuits progress to the highest court, it remains unclear if the Court would rule in favor of the Supremacy Clause or find a path to continue its protection of a “states’ rights” approach to controversial legal debates.

Perhaps our most surprising finding was that despite state-level policy debates and federal lawsuits, we found very little awareness of SAPA among Missouri’s gun owners. Moreover, we found a sense of ambivalence towards the policy’s potential effects. Given the human toll of gun injury and death in Missouri and in the nation more broadly, Missouri may wish to take a second look at SAPA — especially if there is a desire for policy to be both citizen informed and for citizens to be informed about the policies on which their lives depend.

## Figures and Tables

**Appendix Table 1:**
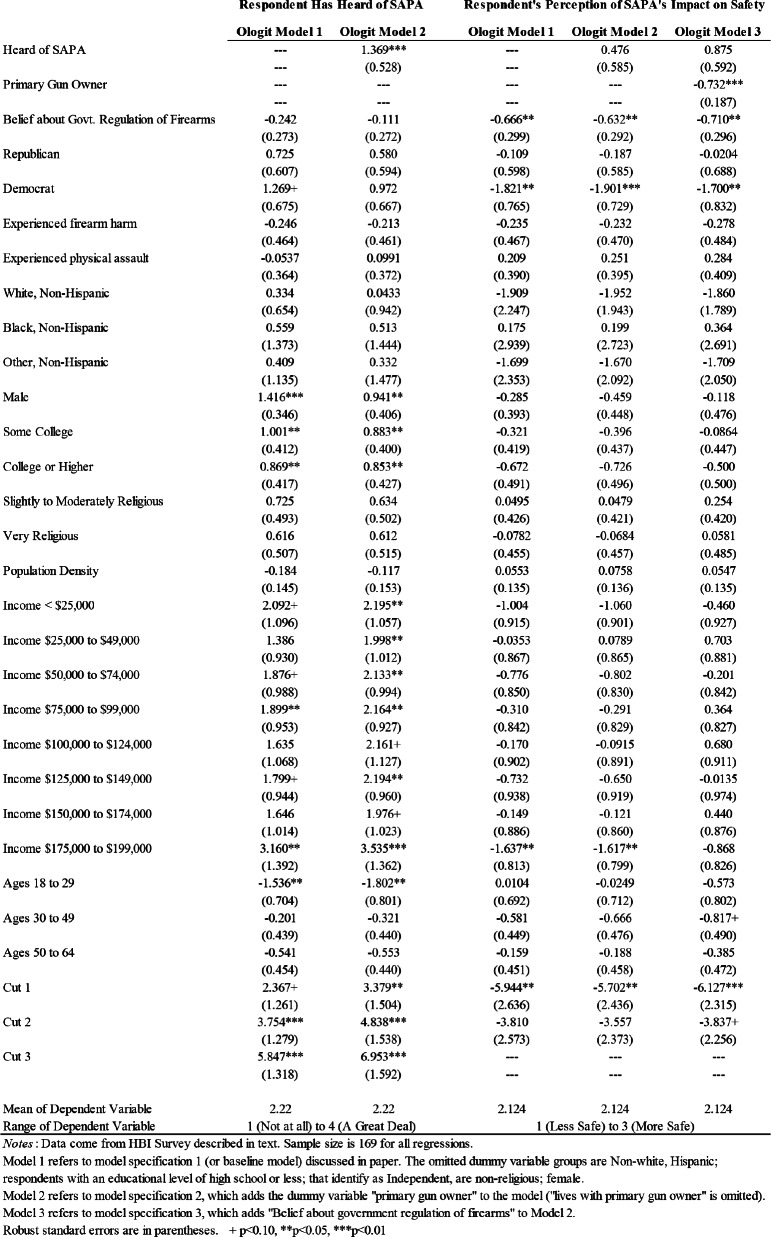
Ordered Logit Results Regarding SAPA Policy

**Appendix Table 2:**
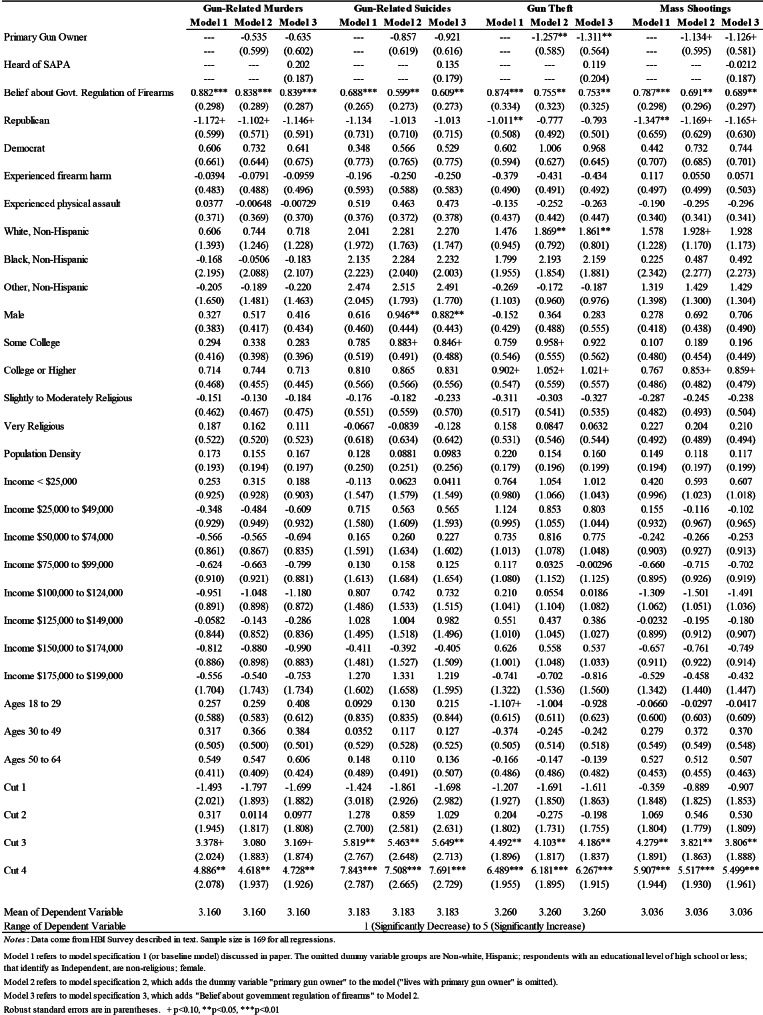
Ordered Logit Results Regarding Respondent’s Belief that SAPA will Impact:

## Note

The authors have no conflicts to dislose.
